# A simplified method for therapeutic drug monitoring of mitotane by gas chromatography‐electron ionization‐mass spectrometry

**DOI:** 10.1002/bmc.4776

**Published:** 2020-01-28

**Authors:** Motozumi Ando, Masaki Hirabatake, Hisateru Yasui, Shoji Fukushima, Nobuyuki Sugioka, Tohru Hashida

**Affiliations:** ^1^ Faculty of Pharmaceutical Sciences Kobe Gakuin University, Kobe Japan; ^2^ Department of Pharmacy Kobe City Medical Center General Hospital, Kobe, Japan; ^3^ Department of Medical Oncology Kobe City Medical Center General Hospital, Kobe, Japan

**Keywords:** adrenal cortical carcinoma, electron ionization, gas chromatography, mitotane, therapeutic drug monitoring

## Abstract

Mitotane is a key drug for the treatment of adrenal cortical carcinoma. Due to its narrow therapeutic window, 14–20 μg/mL, monitoring its concentration is crucially important. In this study, a simplified method for measuring mitotane in plasma using gas chromatography‐electron ionization‐mass spectrometry (GC‐EI‐MS) was developed. Through deproteination and liquid–liquid extraction, mitotane and an internal standard (IS) were extracted from plasma samples. GC‐EI‐MS yielded retention times of 8.2 and 8.7 min, for mitotane and the IS, respectively, with a total run time of 12 min. Selectivity and intra‐/inter‐batch accuracy and precision analyses provided a lower limit of quantification of 0.25 μg/mL, and a calibration curve between 0.25 and 40 μg/mL had good linearity (coefficient of determination = 0.992). The matrix effect factor and percent recovery of the method had good precision. Additionally, long‐term sample stability was observed below 4°C. In a clinical setting, mitotane levels in plasma from an adrenal cortical carcinoma patient were within calibration range. Therefore, this simplified method can be applied to routine therapeutic drug monitoring of mitotane, which may contribute to improved treatment of adrenal cortical carcinoma.

## 1 INTRODUCTION

Mitotane, a key drug for the treatment of adrenal cortical carcinoma (ACC), has a narrow therapeutic window between 14 and 20 μg/mL; therefore, it is desirable to routinely perform therapeutic drug monitoring (TDM). Although several quantitative methods for enantiomers and major metabolites of mitotane have been developed (Cantillana, Lindström, Eriksson, Brandt, & Bergman, 2009; Inouye, Mio, & Sumino, 1987), the lack of antitumor effects of a major metabolite, 1,1‐(*o*,*p*'‐dichlorodiphenyl) acetic acid, was recently demonstrated (Hescot et al., 2014), suggesting the low importance of TDM of these compounds compared to mitotane. Therefore, a simplified and rapid quantitative method for TDM of mitotane is clinically desired, compared to those of metabolites and chiral bodies.

Although validated liquid chromatography (LC) methods for the measurement of blood (including plasma and serum) mitotane levels have been abundantly reported (Andersen, Warren, Nome, Vesterhus, & Slørdal, 1995; Benecke, Vetter, & De Zeeuw, 1987; De Francia et al., 2006; Garg, Sakoff, & Ackland, 2011; Moolenaar, Niewint, & Oei, 1977; Sinsheimer et al., 1996), gas chromatography (GC) methods are rare (Inouye et al., 1987), despite GC being a major analytical method along with LC. Inouye et al. (1987), previously reported a measurement method for plasma mitotane levels using GC‐electron ionization‐mass spectrometry (GC‐EI‐MS); however, this method required a 1 h heating of plasma‐absorbed filter paper to liberate mitotane. Considering the fast‐paced clinical requirements, these conditions are not applicable for routine TDM of mitotane. Furthermore, a measurement method involving a simplified sample preparation procedure has been recently reported by Feliu et al. (2017); however, this method involves the use of an ultra‐high performance LC (UPLC) system, which may currently be less common than LC or GC systems, although UPLC may be more available in future decades. In addition, this procedure does not involve the use of an internal standard (IS), which creates the difficulty of judging whether the observation of a low mitotane peak is due to a low rate of extraction or a low presence of mitotane. Therefore, for the implementation of routine and on‐site analyses, we determined that a validated method using a common chromatography system, an internal reference, and a simplified and user‐friendly sample preparation procedure was required.

In this study, we aimed to develop a widely usable method for the measurement of plasma mitotane levels by GC‐EI‐MS involving a simplified pretreatment procedure in order to facilitate routine TDM of mitotane.

## 2 EXPERIMENTAL

### 2.1 Materials

Details are provided in Supporting Information.

### 2.2 Samples for the calibration curve and quality control

The concentrations of mitotane in pooled human plasma for the calibration curve were 0.25, 0.5, 1, 10, 20, 30, and 40 μg/mL. Quality control (QC) samples (*n* = 5 for each concentration) for testing intra‐ and inter‐batch variability were 0.25, 20, and 40 μg/mL.

### 2.3 Sample pretreatment

For deproteination, 40 μL of IS (12.5 μg/mL)‐containing methanol was added to each patient plasma or prepared plasma sample used for the calibration curve and QC. To each sample, 150 μL of ethyl acetate was added, followed by vortexing for 10 min. The samples were then centrifuged at 10,000 rpm for 5 min, and the supernatants (120 μL) were collected for GC‐EI‐MS analysis.

### 2.4 Instrumentation

A GC‐EI‐MS (GCMS‐QP2010 Ultra, Shimadzu Corp., Kyoto, Japan) instrument, with a fused‐silica capillary column (0.25 ID, 30 m; Agilent Technologies Inc., Santa Clara, CA, USA) was used. See the Supporting Information for details on analysis conditions.

### 2.5 Data analysis

The slope and intercept of the calibration curve were calculated by weighted least‐squares method (1/*x* ^2^; *x* = concentration) using free software, R (R Core Team, 2018).

### 2.6 Intra‐ and inter‐batch accuracy and precision

Intra‐ and inter‐batch variability was determined by analyzing five replicate QC samples on the same day and over 3 days, respectively.

### 2.7 Matrix effect factor and percent recovery

Details are provided in Supporting Information.

### 2.8 Stability

Details are provided in Supporting Information.

### 2.9 Validation procedure

The method was validated through a reference of international recommendations reported by the US Food and Drug Administration (n.d.) and Viswanathan et al. (2007).

## 3 RESULTS AND DISCUSSION

### 3.1 Method validation

#### 3.1.1 Selectivity

Two major chromatographic peaks with retention times of 8.2 and 8.7 min, respectively, were observed while the total runtime was 12 min (Figure S1). Based on their mass spectra, these peaks were identified as mitotane and the IS, respectively (Figure S2). In testing selectivity, plasma containing 0.25 μg/mL of mitotane showed a peak having a signal‐to‐noise ratio ˃ 5 for six individual blank plasma samples (Figure S3); no interference from the IS occurred (Figure S3).

#### 3.1.2 Intra‐ and inter‐batch variability

**Figure 1:**
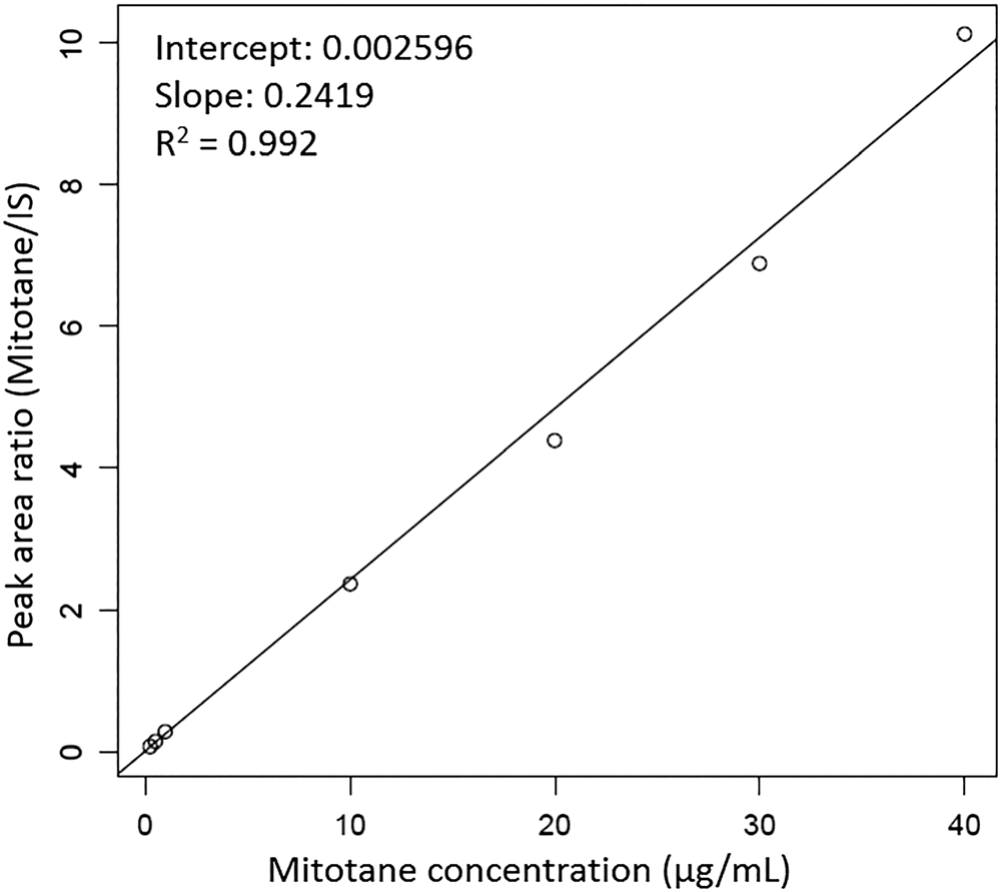
Representative calibration curve generated using an internal standard method. Line‐fitting was achieved using the weighted least‐squares method

As shown in Table 1, acceptable intra‐ and inter‐batch variability was observed. Good linearity of the calibration curve (coefficient of determination = 0.992) was also confirmed between 0.25 and 40 μg/mL (Figure FIGURE 1). Based on selectivity, calibration curve linearity, and variability, 0.25 μg/mL was established as the lower limit of quantification (LLOQ) of the method. It should be noted that the calibration range and LLOQ obtained are superior to those of previously reported methods (Andersen et al., 1995; Benecke et al., 1987; De Francia et al., 2006; Feliu et al., 2017; Garg et al., 2011; Inouye et al., 1987; Mornar, Sertić, Turk, Nigović, & Koršić, 2012).

**Table 1:** Intra‐ and inter‐batch variability of mitotane at low, mid, and high concentration

	Intra‐batch variability (*n* = 5)		Inter‐batch variability (*n* = 15)	
Concentration (μg/mL)	Mean accuracy (%)	Precision (%)	Mean accuracy (%)	Precision (%)
0.25	92.0	7.8	101.6	9.5
20	91.4	4.4	92.2	4.3
40	96.9	5.0	101.1	3.6

#### 3.1.3 Matrix effect factor and percent recovery

The matrix effect factor and percent recovery had acceptable precision. An unexpectedly large recovery rate (mean = 127.5%) was observed at 40 μg/mL (Table S1), although this did not have a significant effect on calibration linearity or intra‐/inter‐batch variability.

#### 3.1.4 Stability

Acceptable accuracy and precision were observed for all stability‐test conditions, with the exceptions of 40 μg/mL mitotane samples stored at 25°C for 14 and 28 days (Table S2). Therefore, if sample storage is required, it should be performed at ≤4°C.

### 3.2 Clinical applicability of the established method

Clinical samples were obtained from an ACC patient, and the plasma mitotane levels were quantitated (Figure S1). The average mitotane concentration was 10.7 μg/mL (median = 9.1 μg/mL), with minimum and maximum detected levels of 8.6 and 14.5 μg/mL, respectively (Table S3). All clinical mitotane levels were within the calibration range of the established method. Thus, this newly developed, validated, and simplified quantitative method for mitotane in plasma is considered applicable to routine TDM, because our method requires only 15 min for deproteination compared to the 1 h pretreatment time required in the method by Inouye et al. (1987). We believe that our method with a simplified and sped‐up procedure for sample pretreatment may be more compatible with a clinical setting.

## 4 CONCLUSIONS

A simplified, validated, and clinically applicable GC‐EI‐MS method for mitotane in plasma was developed and determined to be applicable for routine TDM. Treatment involving TDM of mitotane will likely contribute to improved success of treatment of ACC patients.

## CONFLICT OF INTEREST

The authors declare no conflicts of interest.

## Supporting information

Figure S1. Entire chromatograms of *m/z* 235. (a) Blank plasma; (b) plasma spiked with 0 μg/mL of mitotane; (c) plasma spiked with 0.25 μg/mL (LLOQ) of mitotane; (d) plasma spiked with 40 μg/mL (upper limit of quantification) of mitotane; (e) representative plasma sample from an ACC patient treated with mitotane.
Figure S2. Mass spectra of mitotane (a) and the IS (b) with chromatographic elution times of 8.2 min and 8.7 min, respectively.
Figure S3. Selected ion (i.e., *m/z* 235) monitoring chromatograms of blank plasma (left panels), plasma spiked with 0.25 μg/mL of mitotane (middle panels), and plasma treated with the IS‐containing acetonitrile (right panels). The specificity of the method for mitotane and the IS was confirmed using six individual plasma samples.
Table S1. Matrix effect factor and percent recovery of mitotane at low and high concentrations.
Table S2. Results of short‐, mid‐, and long‐term storage stability.
Table S3. Clinical plasma mitotane levels.

## References

[bmc4776-bib-0001] Andersen, A. , Warren, D. J. , Nome, O. , Vesterhus, L. , & Slørdal, L. (1995). A high‐pressure liquid chromatographic method for measuring mitotane [1,1‐(*o,p’*‐Dichlorodiphenyl)‐2,2‐dichloroethane] and its metabolite 1,1‐(*o,p’*‐Dichlorodiphenyl)‐2,2‐dichloroethene in plasma. Therapeutic Drug Monitoring, 17(5), 526–531. 10.1097/00007691-199510000-00015 8585118

[bmc4776-bib-0002] Benecke, R. , Vetter, B. , & De Zeeuw, R. A. (1987). Rapid micromethod for the analysis of mitotane and its metabolite in plasma by gas chromatography with electron‐capture detection. Journal of Chromatography, 417(2), 287–294. 10.1016/0378-4347(87)80122-6 3654882

[bmc4776-bib-0003] Cantillana, T. , Lindström, V. , Eriksson, L. , Brandt, I. , & Bergman, A. (2009). Interindividual differences in *o,* *p’*‐DDD enantiomer kinetics examined in Göttingen minipigs. Chemosphere, 76(2), 167–172. 10.1016/j.chemosphere.2009.03.050 19394667

[bmc4776-bib-0005] De Francia, S. , Pirro, E. , Zappia, F. , De Martino, F. , Sprio, A. E. , Daffara, F. , … Ghezzo, F. (2006). A new simple HPLC method for measuring mitotane and its two principal metabolites Tests in animals and mitotane‐treated patients. Journal of Chromatography. B, Analytical Technologies in the Biomedical and Life Sciences, 837(1–2), 69–75. 10.1016/j.jchromb.2006.04.005 16698327

[bmc4776-bib-0006] Feliu, C. , Cazaubon, Y. , Guillemin, H. , Vautier, D. , Oget, O. , Millart, H. , … Djerada, Z. (2017). Therapeutic drug monitoring of mitotane: Analytical assay and patient follow‐up. Biomedical Chromatography: BMC, 31(11). e3993 10.1002/bmc.3993 28432798

[bmc4776-bib-0008] Garg, M. B. , Sakoff, J. A. , & Ackland, S. P. (2011). A simple HPLC method for plasma level monitoring of mitotane and its two main metabolites in adrenocortical cancer patients. Journal of Chromatography. B, Analytical Technologies in the Biomedical and Life Sciences, 879(23), 2201–2205. 10.1016/j.jchromb.2011.06.001 21719363

[bmc4776-bib-0009] Hescot, S. , Paci, A. , Seck, A. , Slama, A. , Viengchareun, S. , Trabado, S. , … Lombès, M. (2014). The lack of antitumor effects of *o,* *p'*DDA excludes its role as an active metabolite of mitotane for adrenocortical carcinoma treatment. Hormones & Cancer, 5(5), 312–323. 10.1007/s12672-014-0189-7 25026941 PMC5127823

[bmc4776-bib-0010] Inouye, M. , Mio, T. , & Sumino, K. (1987). Use of GC/MS/SIM for rapid determination of plasma levels of *o,* *p’*‐DDD, *o,* *p’*‐DDE and *o,* *p’*‐DDA. Clinica Chimica Acta; International Journal of Clinical Chemistry, 170(2–3), 305–314. 10.1016/0009-8981(87)90141-0 3436064

[bmc4776-bib-0011] Moolenaar, A. J. , Niewint, J. W. , & Oei, I. T. (1977). Estimation of *o,* *p’*‐DDD in plasma by gas‐liquid chromatography. Clinica Chimica Acta; International Journal of Clinical Chemistry, 76(2), 213–218. 10.1016/0009-8981(77)90098-5 862195

[bmc4776-bib-0012] Mornar, A. , Sertić, M. , Turk, N. , Nigović, B. , & Koršić, M. (2012). Simultaneous analysis of mitotane and its main metabolites in human blood and urine samples by SPE‐HPLC technique. Biomedical Chromatography: BMC, 26(11), 1308–1314. 10.1002/bmc.2696 22259022

[bmc4776-bib-0004] R Core Team . (2018). R: A language and environment for statistical computing. Vienna, Austria. Retrieved from https://www.r-project.org/

[bmc4776-bib-0013] Sinsheimer, J. E. , Counsell, R. E. , Cai, W. , Gopalaswamy, R. , Mahalakshmi, P. , Piñeiro‐Sánchez, M. L. , … Schteingart, D. E. (1996). Gas chromatographic‐electron capture determination of 2,4′‐dichlorodiphenylacetic acid from in‐vitro adrenal transformations of mitotane and its analogs. Journal of Pharmaceutical and Biomedical Analysis, 14(7), 861–866. 10.1016/0731-7085(96)01729-3 8809711

[bmc4776-bib-0007] US Food and Drug Administration . (n.d.). Bioanalytical method validation. Retrieved from https://www.fda.gov/files/drugs/published/Bioanalytical-Method-Validation-Guidance-for-Industry.pdf

[bmc4776-bib-0014] Viswanathan, C. T. , Bansal, S. , Booth, B. , DeStefano, A. J. , Rose, M. J. , Sailstad, J. , … Weiner, R. (2007). Quantitative bioanalytical methods validation and implementation: best practices for chromatographic and ligand binding assays. Pharmaceutical Research, 24(10), 1962–1973. 10.1007/s11095-007-9291-7 17458684

