# Supplementary material for: A simplified method for therapeutic drug monitoring of mitotane by gas chromatography‐electron ionization‐mass spectrometry
Source: Biomed Chromatogr. 2020 Jan 28;34(3):e4776. doi: 10.1002/bmc.4776 (PMC7064981; doi:10.1002/bmc.4776)
Supplement: Supplementary file 1 — Figure S1. Entire chromatograms of m/z 235. (a) Blank plasma; (b) plasma spiked with 0 μg/mL of mitotane; (c) plasma spiked with 0.25 μg/mL (LLOQ) of mitotane; (d) plasma spiked with 40 μg/mL (upper limit of quantification) of mitotane; (e) representative plasma sample from an ACC patient treated with mitotane. Figure S2. Mass spectra of mitotane (a) and the IS (b) with chromatographic elution times of 8.2 min and 8.7 min, respectively. Figure S3. Selected ion (i.e., m/z 235) monitoring chromatograms of blank plasma (left panels), plasma spiked with 0.25 μg/mL of mitotane (middle panels), and plasma treated with the IS‐containing acetonitrile (right panels). The specificity of the method for mitotane and the IS was confirmed using six individual plasma samples. Table S1. Matrix effect factor and percent recovery of mitotane at low and high concentrations. Table S2. Results of short‐, mid‐, and long‐term storage stability. Table S3. Clinical plasma mitotane levels. [file BMC-34-e4776-s001.docx]

**
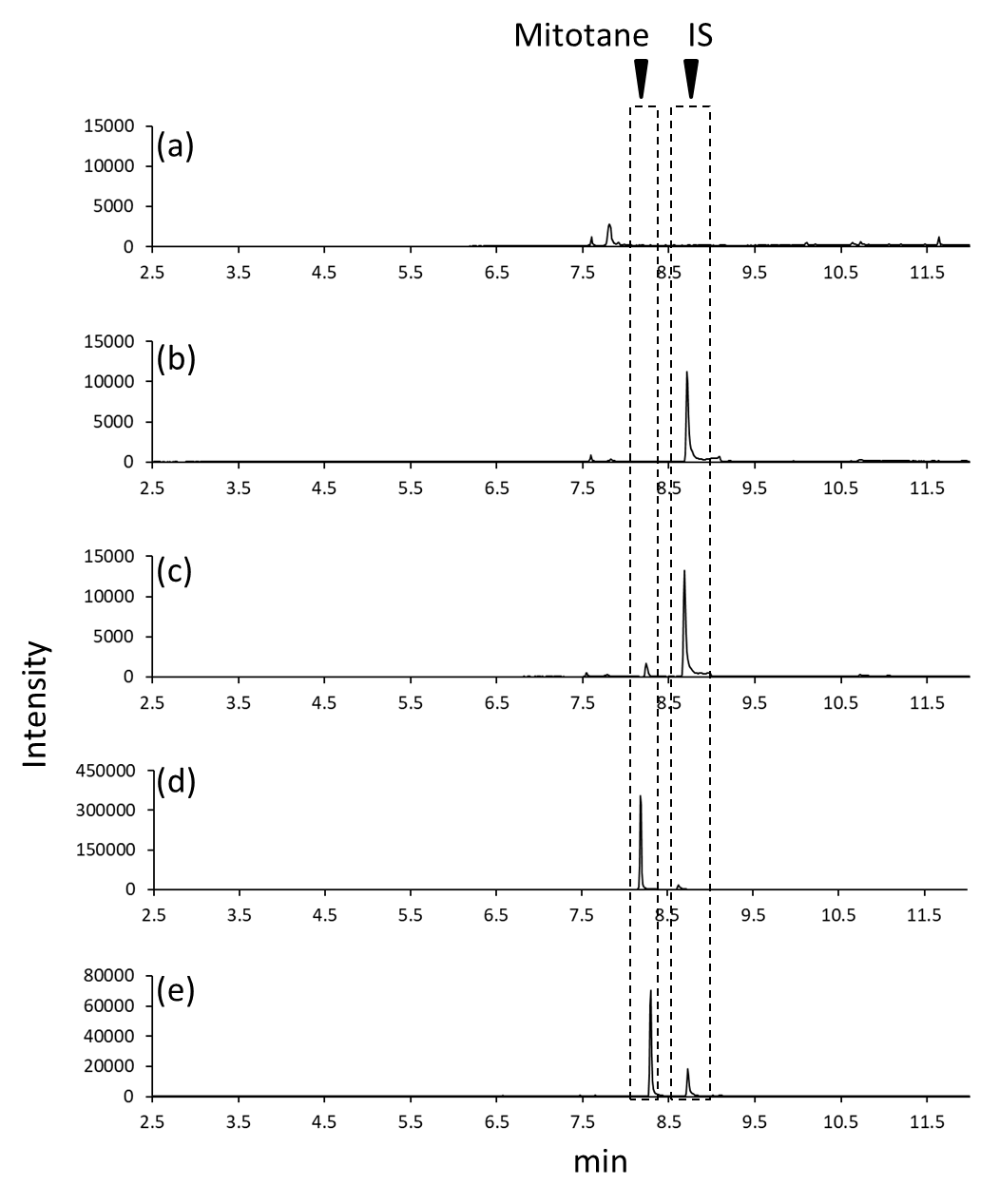
Supporting Information**

**Figure S1**

Figure S1. Entire chromatograms of *m/z* 235. (a) Blank plasma; (b) plasma spiked with 0 µg/mL of mitotane; (c) plasma spiked with 0.25 µg/mL (LLOQ) of mitotane; (d) plasma spiked with 40 µg/mL (upper limit of quantification) of mitotane; (e) representative plasma sample from an ACC patient treated with mitotane.


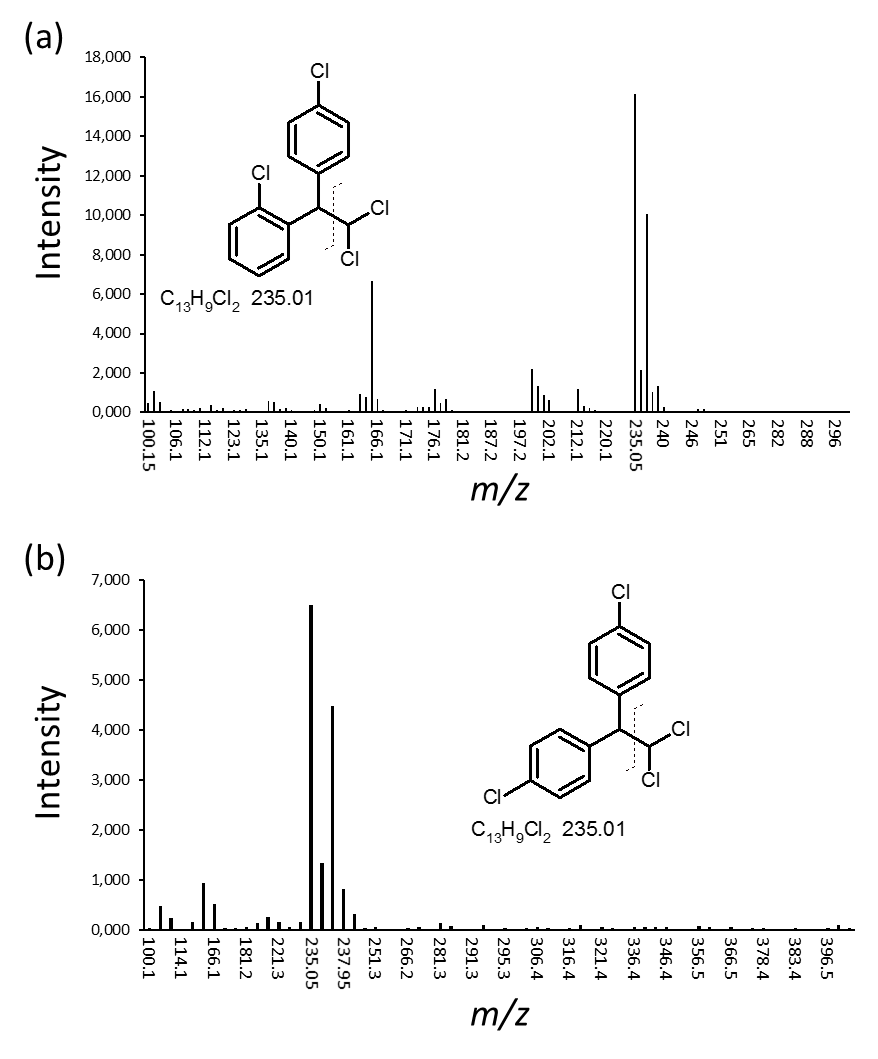


**Figure S2**

Figure S2. Mass spectra of mitotane (a) and the IS (b) with chromatographic elution times of 8.2 min and 8.7 min, respectively.


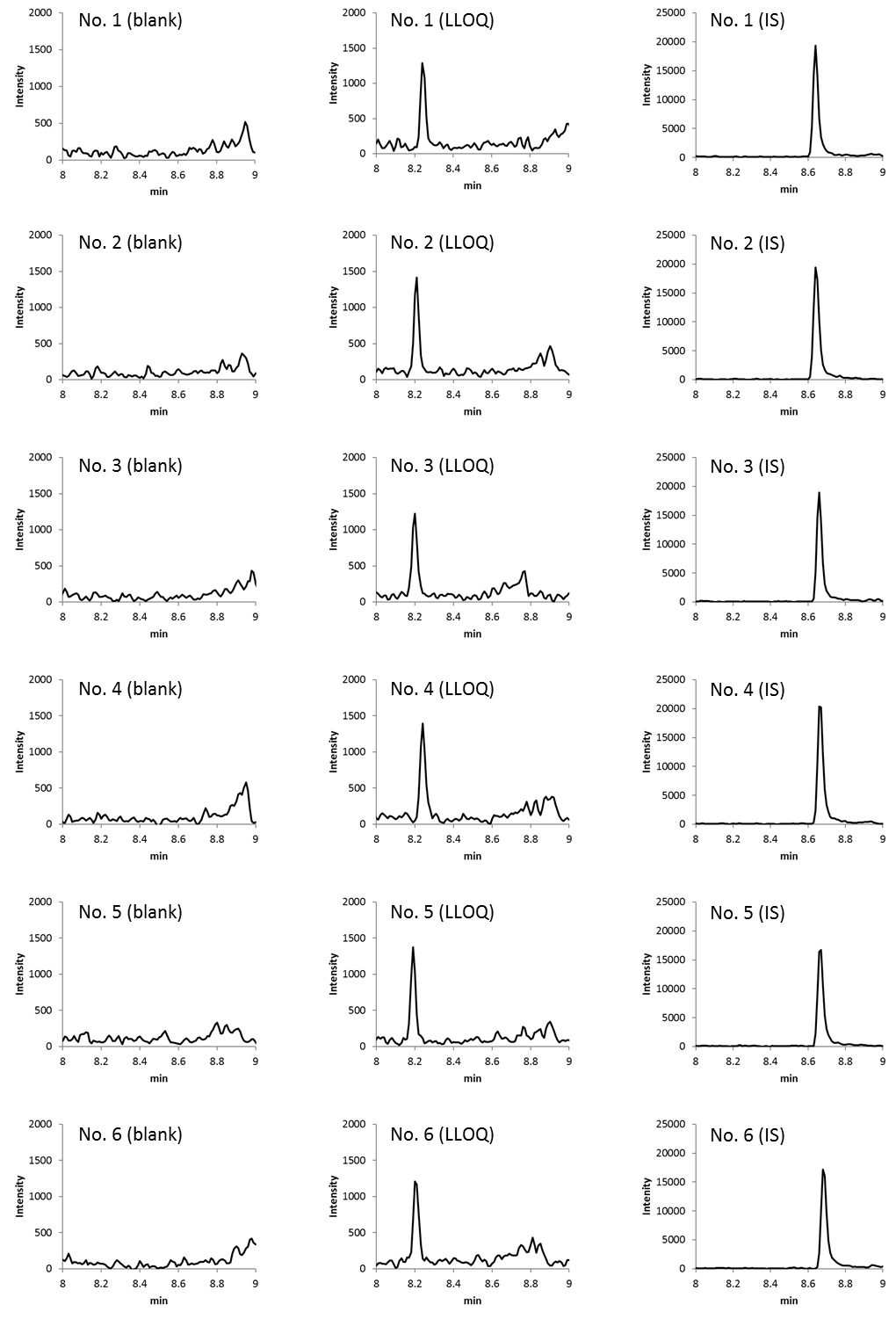


**Figure S3**

Figure S3. Selected ion (i.e., *m/z* 235) monitoring chromatograms of blank plasma (left panels), plasma spiked with 0.25 µg/mL of mitotane (middle panels), and plasma treated with the IS-containing acetonitrile (right panels). The specificity of the method for mitotane and the IS was confirmed using 6 individual plasma samples.

| Table S1. Matrix effect factor and percent recovery of mitotane at low and high concentrations. | | | | | |
| --- | --- | --- | --- | --- | --- |
|  | Matrix effect (n = 5) | |  | Recovery (n = 5) | |
| Conc. (µg/mL) | Mean ± S.D.^a^ | Precision (%) |  | Mean ± S.D. (%) | Precision (%) |
| 0.25 | 1.24 ± 0.11 | 8.9 |  | 100.0 ± 8.31 | 8.3 |
| 40 | 1.31 ± 0.10 | 6.4 |  | 127.5 ± 4.5 | 3.6 |
| ^a^standard deviation | | | | | |

| Table S2. Results of short-, mid-, and long-term storage stability. | | | | | | | |
| --- | --- | --- | --- | --- | --- | --- | --- |
| Conc. (μg/mL) | Accuracy (%) | | |  | Precision (%) | | |
|  | 24 h | 14 days | 28 days |  | 24 h | 14 days | 28 days |
| 0.25 | 25°C | | |  | 25°C | | |
|  | 96.6 | 92.4 | 80.2 |  | 2.1 | 2.8 | 3.9 |
|  | 4°C | | |  | 4°C | | |
|  | 103.0 | 112.4 | 97.7 |  | 4.2 | 6.9 | 5.4 |
|  | -30°C | | |  | -30°C | | |
|  | 101.1 | 119.1 | 90.7 |  | 2.6 | 7.6 | 8.0 |
| 40 | 25°C | | |  | 25°C | | |
|  | 105.6 | 82.4 | 84.9 |  | 3.2 | 12.4 | 1.8 |
|  | 4°C | | |  | 4°C | | |
|  | 109.0 | 96.4 | 96.6 |  | 0.9 | 9.6 | 7.1 |
|  | -30°C | | |  | -30°C | | |
|  | 111.0 | 106.1 | 96.5 |  | 2.3 | 13.7 | 7.0 |

| Table S3. Clinical plasma mitotane levels. | | | | | | |
| --- | --- | --- | --- | --- | --- | --- |
| Mean (μg/mL) | S.D.^a^ | Median (μg/mL) | Min.^b^ (μg/mL) | Max.^c^ (μg/mL) |  |  |
| 10.7 | 3.3 | 9.1 | 8.6 | 14.5 |  |  |
| ^a^standard deviation; ^b^minimum mitotane concentration; ^c)^observed maximum mitotane concentration | | | | |  |  |

**Experimental**

**Materials**

Mitotane was purchased from Sigma-Aldrich (St. Louis, MO, USA). 2,2-bis(4-chlorophenyl)-1,1-dichloroethane was used as an internal standard (IS) and purchased from Tokyo Chemical Industry Co., Ltd. (Tokyo, Japan). Methanol was purchased from Nacalai Tesque, Inc. (Kyoto, Japan). Pooled human plasma was purchased from Cosmo Bio Co. Ltd. (Tokyo, Japan), and individual human plasma samples were obtained from BioIVT (Westbury, NY, USA). Ethyl acetate was purchased from Fujifilm Wako Pure Chemical Corp. (Osaka, Japan).

**Instrumentation**

GC-EI-MS analysis was conducted using a temperature program: 60°C for 1 min, increase from 60 to 210°C at 100°C/min, increase from 210 to 270°C at 10°C/min, and hold at 320°C for 1.5 min. Helium was used as a carrier gas at a constant flow of 2.0 mL/min. The injection port and detector were 260 and 280°C, respectively. Injection (1 µL) was performed in splitless mode. Electron ionization mass spectrometry (70 eV) using selected ion monitoring mode (*m/z* 235) was used to detect both mitotane and the IS.

**Matrix effect factor and percent recovery**

Percent recovery was evaluated by comparing the mean peak areas of mitotane in the specimens after extraction, before and after mitotane spiking. The matrix effect factor was calculated by comparing the mean peak area of mitotane in the extracted specimens with that in a mixture of methanol and ethyl acetate (50:50). Percent recoveries and matrix effect factors were calculated at both 0.25 and 40 µg/mL of mitotane.

**Stability**

QC samples at the LLOQ and 40 µg/mL (n = 3, for each temperature and storage time) were stored at 25, 4, and -30°C. To assess short-term storage stability, accuracy and precision were determined after 24 h. To assess the mid- and long-term storage stability, samples were evaluated after 14 and 28 days, respectively.
